# Effects of thermal treatments on 10 major phenolics and their antioxidant contributions in *Acer truncatum* leaves and flowers

**DOI:** 10.1098/rsos.180364

**Published:** 2018-06-27

**Authors:** Lingguang Yang, Peipei Yin, Chi-Tang Ho, Miao Yu, Liwei Sun, Yujun Liu

**Affiliations:** 1 National Engineering Laboratory for Tree Breeding, College of Biological Sciences and Biotechnology, Beijing Forestry University, Haidian District, Beijing, People's Republic of China; 2 Department of Food Science, Rutgers University, New Brunswick, NJ, USA

**Keywords:** *Acer truncatum* leaves and flowers, thermal treatment, phenolics, antioxidant contribution, UPLC-DAD-QTOF-MS/MS analysis

## Abstract

This study aimed to investigate effects of thermal treatments on major phenolics and their antioxidant contributions in *Acer truncatum* leaves and flowers (ATL and ATF, respectively). With ultra performance liquid chromatography-diode array detector-quadrupole time-of-flight-mass spectrometer/mass spectrometer, phenolic compositions of ATF were first characterized and compared with those of ATL. An optimized high performance liquid chromatography fingerprint was then established, and 10 major phenolics existing in both ATL and ATF were quantified. Gallic acid derivatives and flavonol-3-*O*-glycosides were found to be their dominant phenolic constituents, with the former being key constituents which was affected by thermal treatments and further influencing the variations of total phenols. Moreover, the mechanism underlining the changes of phenolics in ATL and ATF by the treatments was characterized as a thermolhydrolysis process. During thermal treatments, polymerized gallotannins were hydrolysed to 1,2,3,4,6-pentakis-*O*-galloyl-β-d-glucose, ethyl gallate and gallic acid, resulting in more than fivefold and twofold increase of their contents in ATL and ATF, respectively. By contrast, contents and antioxidant contributions of flavonol-3-*O*-glycosides gradually decreased during the process.\absbreak Overall, this is, to our knowledge, the first report on the effects of thermal treatments on phenolics and their antioxidant contributions in ATL and ATF, and the three gallic acid derivatives with potentially higher bioactivity could be efficiently achieved by thermal treatments.

## 1. Introduction

In order to maintain health benefits, compounds of interest in food products need to withstand processing, to be released from the food matrix and to be bio-accessible in the gastrointestinal tract [1]. Therefore, changes undertaken in food processing profoundly affect health benefits of target compounds. Thermal treatment is a major process to convert food to edible form and extend its shelf life [2], and can also break down covalent bonds in insoluble bound phenolics and liberate natural antioxidants from plants [3]. To date, studies have been conducted on how thermal treatment affected phenolic compositions in blueberry [4], elderberry [5] and clove [6], but conflicting results exist regarding its effects in these materials.

*Acer truncatum* is a prominent maple species native to China, Korea and Japan, and is also found in Europe and Northern America [6,7]. After being acknowledged as a new food resource by the Ministry of Health of the People's Republic of China in 2011, the cultivation area of *A. truncatum* has now reached more than 766.7 km² in China. Other than its ornamental function and ability to yield superior vegetable oil, *A. truncatum* leaves (ATL) have long been used as folk medicine and a healthy drink to treat coronary arteriosclerosis, cerebrovascular diseases and angina pectoris in China [8]. Recent research demonstrated that ATL possess various biological functions, such as antioxidant [9], antibacterial [10,11] and antitumour [12,13] effects owing to its high content of phenolics. In our previous studies, we modified an ultrasonic-assisted extraction method with response surface methodology to obtain a high phenolic yield and antioxidant capacity from ATL, and identified 29 phenolics with ultra performance liquid chromatography-quadrupole time-of-flight-mass spectrometer/mass spectrometer (UPLC-QTOF-MS/MS) [14]. Through investigating the seasonal dynamics of phenolics and antioxidant activity, the optimized harvesting period of ATL was also confirmed. Furthermore, several individual phenolics in ATL were subsequently determined, and 1,2,3,4,6-penta-*O*-galloyl-β-d-glucose and quercetin 3-*O*-rhamnoside were identified as its important antioxidant contributors [15].

While a number of researches have been conducted with ATL, *A. truncatum* flowers (ATF) was out of focus. To the best of our knowledge, no investigation has been done concerning phenolics or bioactivity of ATF. Lately, Zhang *et al*. [16] reported that the flowers and leaves of *A. rubrum* contained similar phenolic composition and bioactivity. According to this report, we speculated that ATF might also exhibit similar phenolic compositions to ATL, thus could be considered as a potential functional food resource.

In this study, we aimed to address the impact of thermal treatment on changes of total phenols, overall antioxidant abilities, individual phenolic profiles and their antioxidant contributions in ATL and ATF. This would, to our knowledge, be the first report on effects of thermal treatments on antioxidant contributions as well as phenolics composition of ATL and ATF, and these data should be able to contribute to better use of ATL and ATF.

## 2. Material and methods

### 2.1. Chemicals and plant materials

All authentic standards for high performance liquid chromatography (HPLC) analyses were purchased from National Institutes for Food and Drug Control (Beijing, China) except tannic acid, which was from Sigma Chemical Company (St Louis, MO, USA). Folin–Ciocalteu reagent, 6-hydroxy-2,5,7,8-tetramethylchroman-2-carboxylic acid (Trolox), 2,2′-azobis-(3-ethylbenzothiazoline-6-sulfonic acid) (ABTS), 2,2′-azobis (2-methylpropionamidine) dihydrochloride (AAPH), and fluorescein were purchased from Sigma Chemical Company. HPLC-grade acetonitrile and formic acid were bought from Fisher Scientific (Pittsburgh, PA, USA). Ultra-pure water was prepared using a Milli-Q50 SP Reagent Water System (Millipore Corporation, Billerica, MA, USA). Other reagents (analytical grade) were purchased from Sinopharm Chemical Reagent Company (Beijing, China).

Twenty *Acer truncatum* Bunge trees with similar tree-age and growing environment were randomly selected in Bajia Outskirts Park (GPS coordinates, 40°00'57.21^″^ N, 116°19'43.36^″^ E), Beijing, China, and authenticated by Dr Zhonghua Liu from Beijing Forestry University. Both ATL and ATF were collected on 20 April 2017, which was the florescence period of *A. truncatum*, as well as an optimal season with high phenolic contents and antioxidant activity in ATL [15].

### 2.2. Thermal treatments of *Acer truncatum* leaf and flower extracts

To prepare phenolic extracts, the ultrasonic-assisted extraction procedure previously optimized was conducted [14]. To be specific, 15.30 ml of 66.20% ethanol was added to 1.000 g air-dried and ground ATL or ATF powder, the mixture was then sonicated (KQ-300DE type, Kunshan Ultrasonic Instrument Co., Ltd., Kunshan, China) in a 270 W and 50°C water bath for 30 min. The sonicated mixture was filtered to obtain the supernatant. The extraction process was repeated two more times with the residue, and three supernatants were poured together and diluted to 45.90 ml for further experiments.

For thermal treatment, 10 ml of ATL or ATF extract solutions was placed at 100°C in a sirocco-blasting drying trunk. The treatment time was set at 0.5, 1, 2, 4, 8, 10, 12 or 24 h. After the treatments, all extract solutions were diluted to 10 ml for further experiments.

### 2.3. Characterization of phenolic composition by ultra performance liquid chromatography-diode array detector-quadrupole time-of-flight-mass spectrometer/mass spectrometer analysis

The ultra performance liquid chromatography-diode array detector-quadrupole time-of-flight-mass spectrometer/mass spectrometer (UPLC-DAD-QTOF-MS/MS) system comprised an Acquity UPLC system (Waters, Milford, MA, USA), a DAD detector and a QTOF-MS mass spectrometer (Xevo G2-XS, Waters). The chromatographic separation was performed on a C_18_ reversed phase column (5 µm, 250 × 4.6 mm i.d., Dikma, China) with column temperature set at 30°C. The mobile phase was consisted of water with 0.4% formic acid (v : v) (A) and acetonitrile (B) under the following gradient program: 0–10 min, 10% B; 10–12 min, 10–14% B; 12–40 min, 14% B; 40–80 min, 14–20% B; 80–120 min, 20–23% B. The flow rate was set at 1 ml min^−1^ with an injection volume of 10 µl.

Mass spectra were recorded in the range of *m/z* 50–2000. MS experiments were performed both in positive and negative ionization modes under the following conditions: nitrogen drying gas flow, 10.0 l min^−1^; nebulizer pressure, 45 psi; gas drying temperature, 370°C; capillary and fragmentor voltage, 2.5 kV and MS/MS collision energies, 20 V.

### 2.4. Quantification of individual phenolics by high performance liquid chromatography analysis

HPLC analyses were performed using a Shimadzu HPLC system (Shimadzu, Kyoto, Japan) equipped with two LC-10AT VP pumps: a SPDM20A ultraviolet detector and a SIL-20AC TH autosampler. The reversed phase column, column temperature, solvent system, gradient programme, as well as the flow rate were the same as those in the UPLC analysis described above. Detection wavelength was set at 280 nm to monitor phenols simultaneously.

To determine the phenolic constituents in ATL and ATF with and without thermal treatment, a stock solution of 10 mixed standards (i.e. gallic acid, neochlorogenic acid, ethyl gallate, myricetin-3-*O*-rhamnoside, quercetin-3-*O*-galactoside, quercetin-3-*O*-glucoside, quercetin-3-*O*-arabinopyranoside, 1,2,3,4,6-pentakis-*O*-galloyl-β-d-glucose, quercetin-3-*O*-rhamnoside and kaempferol-3-*O*-rhamnoside) was prepared, and diluted into six concentrations with 66.20% aqueous ethanol (v/v) for linearity assessment. The mixed standard solutions were injected three times under the HPLC condition described above, and the results revealed that all the calibration curves exhibited good linearity (*R*^2^ > 0.998) within the test range.

### 2.5. Measurement of total phenols by Folin–Ciocalteu assay

Total phenols were determined according to the Folin–Ciocalteu method [17] with slight modifications. In brief, 40 µl of 25% Folin–Ciocalteu solution was added to a 96-well plate, followed by addition of a 20 µl standard (10–400 mg l^−1^ gallic acid, *R*^2^ = 0.999), sample or blank (MilliQ water) to designated wells. After blending, 140 µl of 700 mM Na_2_CO_3_ solution was added to each well and the plate was shaken for 5 min at 250 r.p.m. The microplate was then incubated in the dark at 40°C for 30 min, followed by absorbance measurement at 765 nm with a microplate reader (Bio-Rad xMark™ Microplate Absorbance Spectrophotometer, USA). Results were expressed as mg gallic acid equivalent (GAE) 100 g d.w.^−1^ ATL or ATF powder.

### 2.6. Assessment of antioxidant capacities

#### 2.6.1. 2, 2′-diphenyl-1-picrylhydrazyl• scavenging activity

2,2′-diphenyl-1-picrylhydrazyl (DPPH)• scavenging activity was determined by Brand-Williams *et al*. [18] with slight modifications. In brief, 10 µl standard, sample or blank was added to designated wells of a 96-well microplate, followed by addition of 40 µl of 1 mM freshly prepared DPPH solution to each well. Subsequently, 190 µl methanol was added to each well, and the plate was then stood in an orbital shaker setting at 200 r.p.m. for 1 min. After 30 min incubation in the dark at room temperature, absorbance was recorded at 517 nm using the microplate reader. A standard calibration curve of Trolox (0–400 mg l^−1^, *R*^2^ = 0.999) was plotted, and results were expressed as μmol Trolox equivalent (TE) 100 g d.w.^−1^ ATL or ATF powder.

#### 2.6.2. 2, 2′-azobis-(3-ethylbenzothiazoline-6-sulfonic acid)^+^• scavenging activity

ABTS^+^• scavenging capacities were evaluated by using the method of Re *et al*. [19] with slight modifications. ABTS^+^• was generated by the reaction of a 7 mM ABTS aqueous solution with a 2.4 mM K_2_S_2_O_8_ aqueous solution in equivalents, and the mixture was incubated in the dark at room temperature for 12–16 h. Subsequently, the ABTS solution was diluted with methanol to an absorbance of 0.70 ± 0.02 at 734 nm measured using the microplate reader to generate an ABTS working solution. After the working solution was prepared, a 5 µl standard, sample or blank was added to corresponding wells in a 96-well microplate, followed by addition of 200 µl ABTS working solution to each well. After 5 min incubation in the dark at 30°C, the absorbance was measured also at 734 nm. A standard calibration curve of Trolox (0–800 mg l^−1^, *R*^2^ = 0.998) was plotted, and the results were expressed as μmol Trolox equivalent (TE) 100 g d.w.^−1^ ATL or ATF powder.

#### 2.6.3. Oxygen radical absorbance capacity

Oxygen radical absorbance capacity (ORAC) assay was performed with all reagents prepared in a 75 mM phosphate buffer (pH = 7.4) according to a report by Sun *et al.* [20], and direct light was not allowed during the whole process. Briefly, a 25 µl standard (Trolox 5–50 µM, *R*^2^ = 0.997), sample or blank (75 mM phosphate buffer) was mixed with 75 µl fluorescein (0.20 µM) in wells of a 96-well microplate. After 5 min shaking at 250 r.p.m., the plate was incubated in a 37°C-prewarmed oven for 15 min. Subsequently, 100 µl of 37°C-prewarmed AAPH was immediately added to each well with a 12-channel multipipet to initiate the reaction. The fluorescence was recorded every 1.5 min with a multi-functional fluorescence detector (Tecan infinite M200, Swiss) at 37°C for 75 min with an excitation at 530 nm and emission at 485 nm. The net areas under curve (AUC) of samples and standards were calculated by subtracting the AUC of the blank. Results were calculated by comparing the net AUC of each sample with that of the standard. ORAC values were calculated as μmol Trolox equivalents (TE) 100 g d.w.^−1^ ATL or ATF powder.

### 2.7. Data analysis

Each experiment was carried out in triplicate and data were expressed as mean ± standard deviation. The statistical significance was determined by Tukey's HSD test in one-way ANOVA using SPSS software (v. 22.0, SPSS Inc., Chicago, IL, USA). *p* < 0.05 was set to be significant.

## 3. Results and discussion

### 3.1. Characterization and classification of phenolics in *Acer truncatum* leaves and flowers with ultra performance liquid chromatography-diode array detector-quadrupole time-of-flight-mass spectrometer/mass spectrometer and high performance liquid chromatography fingerprint

To address impacts of thermal treatments on ATL and ATF, their detailed phenolic compositions were characterized through UPLC-DAD-QTOF-MS/MS analysis. Identification process of most phenolics in ATL and ATF in this study has been described in our previous study [14]. As for those phenolics first found in *A. truncatum*, the identification was conducted by comparing their ultraviolet spectra, mass spectra and fragment ions to those in the literature and the METLIN database (https://metlin.scripps.edu). For instance, as shown in table 1, compound 20 in ATL with the parent ion at *m/z* 577.1586 was identified as kaempferol-3,7-*O*-dirhamnoside in that it fragmented at 431.0899 with the loss of a rhamnoside moiety (146) and at 285.0413 (corresponding to kaempferol aglycone) with the loss of another rhamnoside moiety (146) [23].

**Table 1.** Phenolic compounds identified in ATL and ATF by UPLC-DAD-QTOF-MS/MS analysis. (‘K’ represents Kolniak-Ostek [21]; ‘Di’ represents Díaz-de-Cerio *et al*. [22]; ‘A’ represents Aguirre-Hernandez *et al*. [23]; ‘P’ represents Petropoulos *et al*. [24]; ‘Do’ represents Dorta *et al*. [25]; ‘ST’ represents those confirmed by the authentic standards while those without a superscript letter was described in Yang *et al*. [14].)

peak no.	retention time (min)	*λ*_max_ (nm)	[M-H]- (*m/z*)	error (PPM)	formula	MS/MS fragments (*m/z*)	identified compounds^a^
in ATL							
1	2.41	258	191.0560	2.1	C_7_H_11_O_6_	127.0394, 111.0446	quinic acid
2	4.01	230, 272	343.0659	−1.7	C_14_H_15_O_10_	169.0134, 125.0234	theogallin
3	4.22	272, 227	169.0131	−3.6	C_7_H_5_O_5_	169.0115, 125.0207	gallic acid^ST^
4	6.84	327	353.0868	−1.4	C_16_H_17_O_9_	191.0545, 179.0329, 135.0441	3-*O*-caffeoylquinic acid
5	7.46	326	353.0870	−0.8	C_16_H_17_O_9_	191.0584, 179.0339, 135.0438	5-*O*-caffeoylquinic acid^ST^
6	10.41	272	285.0612	0.7	C_12_H_13_O_8_	109.0086, 152.9972	uralenneoside
7	11.33	309	337.0948	7.4	C_16_H_17_O_8_	191.0334, 163.0159, 119.0480	*cis*-4-*p*-coumaroylquinic acid
8	12.06	310	337.0911	−3.6	C_16_H_17_O_8_	191.0545, 163.0401, 119.0509	*cis*-5-*p*-coumaroylquinic acid
9^b^	14.41	327	353.0875	0.6	C_16_H_17_O_9_	191.0456, 179.0218, 173.0207, 135.0424	*cis*-4-caffeoylquinic acid^K^
10^b^	14.92	327	353.0863	−2.8	C_16_H_17_O_9_	191.0531, 173.0421, 135.0434	*trans*-4-caffeoylquinic acid^K^
11	18.37	284	289.0707	−1.7	C_15_H_13_O_6_	245.0250, 211.9531	(+)-Catechin
12	26.01	272, 228	197.0450	0.0	C_9_H_9_O_5_	124.0160	ethyl gallate^ST^
13	31.74	278	787.0982	−1.5	C_34_H_27_O_22_	617.0930, 465.0587, 169.0089	1,2,3,6-tetrakis-*O*-galloyl-β-d-glucose
14	33.24	255, 352	593.1560	9.1	C_27_H_29_O_15_	447.1040, 301.0292	quercetin-3,7-*O*-dirhamnoside
15	35.03	261, 353	449.0706	−3.1	C_20_H_17_O_12_	316.0229, 271.0262	myricetin-arabinoside/xylopyranoside
16	37.64	349, 262	463.0865	−2.6	C_21_H_19_O_12_	316.0202, 287.0173	myricetin-3-*O*-rhamnoside^ST^
17	41.07	265, 358	463.0912	7.6	C_21_H_19_O_12_	300.0266, 271.0263	quercetin-3-*O*-galactoside^ST^
18	44.87	262, 357	463.0859	−3.9	C_21_H_19_O_12_	300.0274, 271.0274	quercetin-3-*O*-glucoside^ST^
19^b^	50.02	265, 355	615.1014	4.6	C_28_H_23_O_16_	301.0369, 178.9859, 150.9996	quercetin-galloylhexoside^Di^
20^b^	53.34	265	577.1586	5.0	C_27_H_29_O_14_	285.0413, 431.0899	kaempferol-3,7-*O*-dirhamnoside^A^
21	54.19	257, 357	433.0777	1.4	C_20_H_17_O_11_	300.0252, 271.0225	quercetin-3-*O*-arabinopyranoside^ST^
22	58.81	281	939.1146	4.5	C_41_H_31_O_26_	769.0890, 617.0790, 169.0135	pentagalloyl glucose isomer
23	60.31	280	939.1157	5.6	C_41_H_31_O_26_	769.0925, 617.0801, 169.0155	1,2,3,4,6-pentakis-*O*-galloyl-β-d-glucose^ST^
24	62.25	256, 347	447.1084	0.9	C_25_H_19_O_8_	300.0313, 271.0285	quercetin 3-*O*-rhamnoside^ST^
25	72.22	277	1091.1260	4.2	C_48_H_35_O_30_	939.1131, 769.0892, 617.0669, 169.0144	hexagalloyl glucose
26	79.55	266	431.0970	−1.9	C_21_H_19_O_10_	285.0390, 255.0280	kaempferol-3-*O*-rhamnoside^ST^
27^b^	82.24	266	461.1064	−4.3	C_22_H_21_O_10_	285.0364, 271.0208	kaempferol-3-*O*-glucuronide^P^
in ATF							
1	2.41	258	191.0553	−1.6	C_7_H_11_O_6_	127.0391, 111.0466	quinic acid
2^b^	2.98	274	331.0665	0.0	C_13_H_15_O_10_	169.0134, 125.0228	galloyl glucose^Do^
3	4.22	227, 272	169.0133	−2.4	C_7_H_5_O_5_	125.0216	gallic acid^ST^
4	7.44	326	353.0885	3.4	C_16_H_17_O_9_	191.0547, 179.0321, 135.0416	5-*O*-caffeoylquinic acid^ST^
5	10.47	272	285.0612	0.7	C_12_H_13_O_8_	109.0183, 152.0100	uralenneoside
6	12.06	310	337.0911	−3.6	C_16_H_17_O_8_	191.0545, 163.0401, 119.0509	coumaroylquinic acid
7^b^	14.64	229	595.1291	−1.3	C_26_H_27_O_16_	462.0802, 299.0132	quercetin-3-*O*-pentosylhexoside^A^
8	26.01	228, 273	197.0451	0.5	C_9_H_9_O_5_	124.0155	ethyl gallate^ST^
9^b^	37.25	269, 352	609.1490	5.6	C_27_H_29_O_16_	465.0681, 300.0294	quercetin-3-*O*-rutinoside^A^
10	37.90	349, 262	463.0736	−5.0	C_21_H_19_O_12_	316.0214, 287.0152	myricetin-3-*O*-rhamnoside^ST^
11	41.33	256, 354	463.0875	−0.4	C_21_H_19_O_12_	300.0252, 271.0237	quercetin-3-*O*-galactoside^ST^
12	45.18	253, 349	463.0916	8.4	C_21_H_19_O_12_	300.0291, 271.0265	quercetin-3-*O*-glucoside^ST^
13^b^	50.45	264	593.1544	6.4	C_27_H_29_O_15_	284.0319, 255.0293	kaempferol-3-*O*-(6-*p*-coumaroyl)-glucoside^A^
14	54.35	256, 354	433.0768	−0.7	C_20_H_17_O_11_	300.0264, 271.0249	quercetin-3-*O*-arabinopyranoside^ST^
15^b^	55.75	266, 348	447.0972	10.1	C_21_H_19_O_11_	284.0315, 255.0285	kaempferol-3-*O*-glucoside^A^
16	59.17	227, 279	939.1108	0.4	C_41_H_31_O_26_	769.0939, 617.0818, 169.0163	pentagalloyl glucose isomer
17	60.62	227, 279	939.1108	0.4	C_41_H_31_O_26_	769.0912, 617.0790, 169.0133	1,2,3,4,6-pentakis-*O*-galloyl-β-d-glucose^ST^
18	62.49	256, 350	447.0919	−1.8	C_21_H_19_O_11_	300.0260, 271.0239	quercetin 3-*O*-rhamnoside^ST^
19	79.83	266	431.0976	−0.5	C_21_H_19_O_10_	284.0309, 255.0305	kaempferol-3-*O*-rhamnoside^ST^

^a^Superscript letters in the column ‘identified compounds' represent the references.

^b^Superscript letters in the column ‘peak number’ represent the compounds first identified in ATL and ATF.

**Figure 1.**
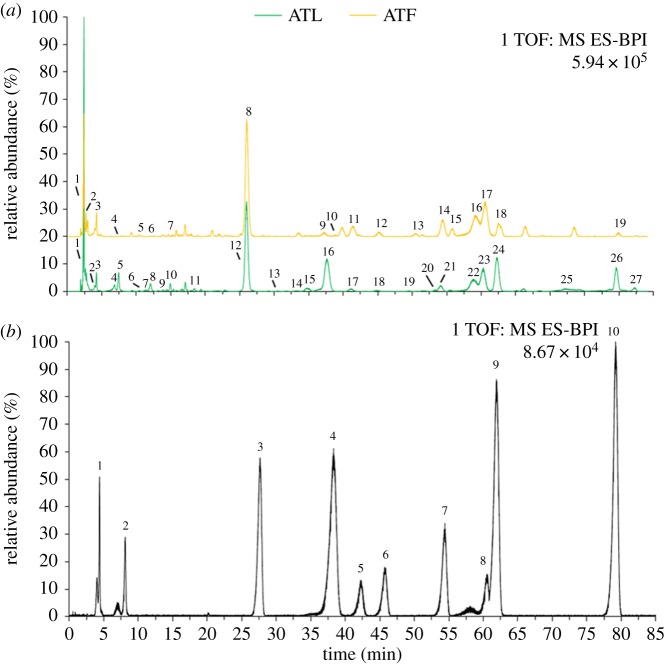
UPLC-DAD-QTOF-MS spectra data of ATL (green curve) and ATF (yellow curve) extracts (*a*), together with the 10 mixed standards (*b*) obtained under the same UPLC conditions. Numbers above the yellow curve correspond to the peak number of phenolics in ATF, while those above the green curve represent the peak number of phenolics in ATL. Numbers 1–10 above the black curve are gallic acid (1), neochlorogenic acid (2), ethyl gallate (3), myricetin-3-*O*-rhamnoside (4), quercetin-3-*O*-galactoside (5), quercetin-3-*O*-glucoside (6), quercetin-3-*O*-arabinopyranoside (7), 1,2,3,4,6-pentakis-*O*-galloyl-β-d-glucose (8), quercetin 3-*O*-rhamnoside (9) and kaempferol-3-*O*-rhamnoside (10).

As shown in table 1 and figure 1*a*, 27 phenolics were identified in unprocessed ATL. Among which, 22 of them had been identified in ATL previously [14] and five of them, namely, *cis*-4-caffeoylquinic acid (9 in ATL profile; similarly hereinafter), *trans*-4-caffeoylquinic acid (10), quercetin-galloylhexoside (19), kaempferol-3,7-*O*-dirhamnoside (20) and kaempferol-3-*O*-glucuronide (27), were identified in *A. truncatum* for the first time (table 1 and the green curve of figure 1*a*). On the other hand, 19 phenolics were identified in ATF (table 1 and the yellow curve of figure 1*a*). As this is the first report on phenolics composition of ATF, all these 19 phenolics were considered to be identified in it for the first time. Nevertheless, 14 of them had been previously identified in ATL, implying that phenolics composition of ATF was similar to that of ATL. In other words, the other five phenolics, namely, galloyl glucose (2 in ATF profile; similarly hereinafter), quercetin-3-*O*-pentosylhexoside (7), quercetin-3-*O*-rutinoside (9), kaempferol-3-*O*-(6-*p*-coumaroyl)-glucoside (13) and kaempferol-3-*O*-glucoside (15), were first identified in *A. truncatum*. Furthermore, all those 10 phenolics identified for the first time in *A. truncatum* in the current study still belonged to gallates, gallotannins, chlorogenic acids and flavonoids [14], which further indicated that phenolic compositions of ATF and ATL consisted of mainly these four groups.

Moreover, there were 14 phenolics existing both in ATL and ATF that were characterized (table 1). Among which, 10 phenolics (i.e. gallic acid, neochlorogenic acid, ethyl gallate, myricetin-3-*O*-rhamnoside, quercetin-3-*O*-galactoside, quercetin-3-*O*-glucoside, quercetin-3-*O*-arabinopyranoside, 1,2,3,4,6-pentakis-*O*-galloyl-β-d-glucose, quercetin-3-*O*-rhamnoside and kaempferol-3-*O*-rhamnoside) exhibited higher intensities in at least one profile (figure 1). Therefore, they were further confirmed by comparing their MS/MS data and retention time with those of the authentic standards (figure 1*b*), and subsequently used as the markers for both ATL and ATF in the following HPLC quantification during the thermal treatments.

**Figure 2.**
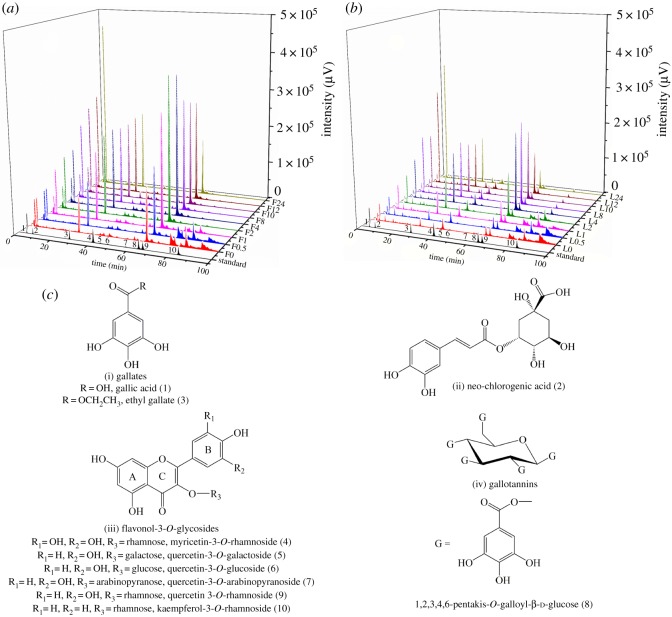
HPLC fingerprints of ATL (*a*) and ATF (*b*) during the thermal treatment as well as the mix standards of the 10 major phenolics, together with the chemical structure of the 10 major phenolics (*c*). Chromatographs of ATF, ATL and mixed standards (black curve) were obtained under the same HPLC conditions. ‘L’ and ‘F’ in the Z axials in (*a*) and (*b*) represent ATL and ATF, respectively, and the number following ‘L’ and ‘F’ represents the thermal treatment duration. Numbers 1–10 in (*a*) and (*b*) correspond to those in (*c*).

As shown in figure 2*a,b*, HPLC spectra of ATL and ATF clearly showed that the 10 phenolics exhibited higher intensities than other compounds during the whole thermal treatment. Therefore, we preliminary considered them as major phenolics in ATL and ATF. Meanwhile, there was a set of less intensive peaks (other than compound 10) eluted out between 75 and 100 min in both profiles, which were difficult to be separated further. For those 10 major phenolics, they could be divided into four categories, i.e. gallates (1 and 3), caffeoylquinic acids (2), flavonol-3-*O*-glycosides (4, 5, 6, 7, 9 and 10) and gallotannins (8) based on their chemical structures (figure 2*c*). As both gallates and gallotannins shared gallic acid as a common subunit, they were regrouped as gallic acid derivatives. In addition, caffeoylquinic acids were recombined with flavonol-3-*O*-glycosides as neochlorogenic acid was the only one in the caffeoylquinic acids category with relatively low intensity. Therefore, we further divided the 10 major phenolics into two main groups, namely, gallic acid derivatives and flavonol-3-*O*-glycosides.

### 3.2. Changes of phenolics under thermal treatment

#### 3.2.1. Changes of 10 major phenolics in *Acer truncatum* leaves

Dynamics of the 10 major phenolics in ATL and ATF during thermal treatments are presented in table 2. In unprocessed ATL, three flavonol-3-*O*-glycosides, namely, myricetin-3-*O*-rhamnoside (1289.51 mg 100 g d.w.^−1^), quercetin-3-*O*-rhamnoside (939.72), and kaempferol-3-*O*-rhamnoside (602.19), had higher contents than three gallic acid derivatives (i.e. 1,2,3,4,6-pentakis-*O*-galloyl-β-d-glucose (470.01), ethyl gallate (227.97) and gallic acid (210.08)) and other identified phenolics, indicating the abundance of flavonol-3-*O*-glycosides, especially flavonol-3-*O*-rhamnosides in ATL (table 2). During thermal treatment, three flavonol-3-*O*-rhamnosides kept relatively steady in the beginning, and dramatically decreased after certain treatment duration. For instance, kaempferol-3-*O*-rhamnoside slightly increased to 109.63% at 2 h, but dramatically decreased to 34.18% at 8 h, and continuously declined to 20.7% at 24 h compared with those in unprocessed ATL (table 2).

**Table 2.** Changes of total phenols (HPLC and Folin–Ciocalteu), together with 10 major phenolics from ATL and ATF for different thermal treatment times. (Results of the contents of 10 major phenolics are expressed as mg individual phenolics 100 g d.w.^−1^, and different letters in each row mean significant difference (*p* < 0.05). n.d., not detected.)

	treatment time (h)								
phenolic compounds	0	0.5	1	2	4	8	10	12	24
in ATL (peak no.)									
*gallic acid derivatives*									
gallic acid (1)	210.08 ± 1.08^i^	224.42 ± 1.19^h^	267.46 ± 1.53^g^	315.34 ± 1.93^f^	410.97 ± 2.74^e^	579.05 ± 4.18^d^	677.66 ± 5.03^c^	1057.79 ± 8.31^b^	1325.74 ± 10.63^a^
ethyl gallate (3)	227.97 ± 1.22^h^	289.31 ± 1.72^g^	499.64 ± 3.50^f^	715.05 ± 5.35^e^	1058.25 ± 8.31^b^	1159.87 ± 9.19^a^	1050.25 ± 8.24^b^	960.68 ± 7.47^d^	998.87 ± 7.80^c^
1,2,3,4,6-pentakis-*O*-galloyl-β-d-glucose (8)	470.01 ± 1.87^h^	588.24 ± 2.08^g^	1011.69 ± 3.89^f^	1862.42 ± 8.54^e^	3171.77 ± 15.80^b^	3357.17 ± 12.83^a^	2878.48 ± 14.52^c^	1907.9 ± 8.46^d^	1037.79 ± 11.93^f^
*caffeoylquinic acid*									
neochlorogenic acid (2)	231.17 ± 1.24^b^	238.31 ± 1.30^a^	232.98 ± 1.26^b^	209.02 ± 1.07^c^	183.82 ± 0.88^d^	171.15 ± 0.79^e^	138.6 ± 0.55^f^	137.73 ± 0.63^f^	137.27 ± 0.72^f^
*flavonol-3-O-glycosides*									
myricetin-3-*O*-rhamnoside (4)	1289.51 ± 12.31^b^	1237.62 ± 9.86^c^	1263.37 ± 14.09^b^	1329.02 ± 10.66^a^	1339.60 ± 10.74^a^	1321.89 ± 13.59^a^	1053.59 ± 8.27^d^	966.12 ± 7.52^e^	766.72 ± 5.80^f^
quercetin-3-*O*-galactoside (5)	99.54 ± 0.50^d^	107.34 ± 0.72^c^	93.59 ± 0.39^e^	127.39 ± 1.23^b^	188.03 ± 1.06^a^	130.88 ± 1.37^b^	99.22 ± 0.42^d^	91.87 ± 0.08^e^	84.38 ± 0.36^f^
quercetin-3-*O*-glucoside (6)	36.06 ± 0.17^c^	35.95 ± 0.04^c^	37.2 ± 0.14^bc^	38.34 ± 0.25^b^	43.21 ± 0.62^a^	31.49 ± 0.09^d^	24.89 ± 0.28^e^	n.d.	n.d.
quercetin-3-*O*-arabinopyranoside (7)	76.03 ± 0.30^cd^	82.28 ± 0.29^a^	77.69 ± 0.13^c^	75.05 ± 0.39^de^	79.47 ± 0.11^b^	73.65 ± 0.62^e^	60.84 ± 0.04^f^	53.58 ± 0.22^g^	34.79 ± 0.05^h^
quercetin 3-*O*-rhamnoside (9)	939.72 ± 4.83^f^	981.07 ± 3.98^d^	1006.04 ± 3.06^c^	966.25 ± 2.92^e^	1087.71 ± 3.34^a^	1038.63 ± 1.17^b^	980.66 ± 2.08^d^	864.86 ± 5.62^g^	589.53 ± 0.73^h^
kaempferol-3-*O*-rhamnoside (10)	602.19 ± 2.12^d^	714.12 ± 3.42^b^	807.38 ± 6.18^a^	660.16 ± 2.07^c^	315.62 ± 5.93^e^	205.85 ± 0.56^f^	193.77 ± 1.83^g^	169.31 ± 0.72^h^	124.54 ± 2.46^i^
TPLC*	4182.29 ± 18.87^i^	4498.67 ± 20.14^h^	5297.05 ± 24.20^f^	6298.04 ± 29.37^d^	7878.44 ± 37.33^b^	8069.64 ± 38.87^a^	7157.96 ± 33.92^c^	6209.83 ± 32.34^e^	5099.63 ± 29.03^g^
TPFC*	7579.56 ± 353.52^b^	7223.35 ± 323.74^b^	7736.01 ± 259.34^ab^	7631.25 ± 283.08^b^	7814.24 ± 287.06^ab^	8333.89 ± 289.75^a^	7459.43 ± 277.69^b^	7859.64 ± 256.12^ab^	7636.14 ± 172.56^b^
TPLC/ TPFC*	55.18	62.28	68.47	82.53	100.82	96.83	95.96	79.01	55.18
in ATF (peak no.)									
*gallic acid derivatives*									
gallic acid (1)	340.01 ± 2.25^i^	353.5 ± 2.14^h^	455.79 ± 3.12^g^	519.28 ± 3.66^f^	555.68 ± 5.51^e^	733.15 ± 3.66^d^	901.04 ± 6.95^c^	989.27 ± 7.72^b^	1665.65 ± 13.59^a^
ethyl gallate (3)	728.91 ± 5.47^e^	905.37 ± 6.99^d^	1354.76 ± 10.88^c^	1589.85 ± 12.91^a^	1564.72 ± 15.32^b^	1553.28 ± 8.83^b^	1559.66 ± 12.65^b^	1580.4 ± 14.83^a^	1596.28 ± 12.97^a^
1,2,3,4,6-pentakis-*O*-galloyl-β-d-glucose(8)	1995.71 ± 15.59^i^	2587.77 ± 19.69^g^	4528.24 ± 33.12^d^	5732.79 ± 51.46^a^	5555.93 ± 40.24^b^	4643.71 ± 33.92^c^	4356.61 ± 35.93^e^	4041.69 ± 19.75^f^	2448.35 ± 13.72^h^
*caffeoylquinic acid*									
neochlorogenic acid (2)	77.82 ± 0.52^a^	77.03 ± 0.38^a^	76.91 ± 0.71^a^	71.69 ± 1.82^b^	71.05 ± 0.25^b^	66.99 ± 0.53^c^	48.56 ± 0.94^d^	48.84 ± 0.47^d^	31.88 ± 0.15^e^
*flavonol-3-O-glycosides*									
myricetin-3-*O*-rhamnoside (4)	182.1 ± 0.87^b^	189.32 ± 0.92^a^	191.92 ± 0.94^a^	190.93 ± 0.63^a^	182.63 ± 1.28^b^	167.86 ± 0.77^c^	161.65 ± 0.73^d^	164.25 ± 0.82^cd^	132.65 ± 0.57^f^
quercetin-3-*O*-galactoside (5)	562.14 ± 4.03^a^	431.23 ± 2.91^c^	419.78 ± 2.81^d^	415.46 ± 2.77^d^	513.67 ± 3.62^b^	346.62 ± 2.19^e^	322.87 ± 1.99^f^	324.39 ± 2.31^f^	264.48 ± 1.50^g^
quercetin-3-*O*-glucoside (6)	90.3 ± 0.51^b^	89.45 ± 0.28^b^	73.74 ± 0.33^d^	70.99 ± 0.46^f^	70.86 ± 0.58^f^	93.71 ± 0.50^a^	86.95 ± 0.51^c^	n.d.	n.d.
quercetin-3-*O*-arabino pyranoside (7)	326.33 ± 0.74^c^	346.52 ± 0.81^a^	338.27 ± 0.37^b^	331.42 ± 1.03^c^	301.75 ± 0.66^d^	247.74 ± 0.49^f^	229.46 ± 0.43^g^	222.74 ± 1.18^g^	146.72 ± 0.26^h^
quercetin 3-*O*- rhamnoside (9)	329.45 ± 0.75^a^	329.01 ± 1.32^a^	243.66 ± 0.48^b^	246.88 ± 0.49^b^	242.54 ± 0.47^b^	212.75 ± 0.53^c^	206.56 ± 0.82^cd^	202.91 ± 0.31^d^	167.24 ± 0.38^f^
kaempferol-3-*O*-rhamnoside (10)	731.92 ± 2.12^b^	913.25 ± 4.74^a^	458.85 ± 1.38^c^	67.16 ± 0.32^d^	34.22 ± 0.24^e^	33.82 ± 0.18^e^	32.02 ± 0.60^f^	31.72 ± 0.09^f^	27.84 ± 0.15^g^
TPLC*	5364.69 ± 31.15^h^	6222.44 ± 36.49^g^	8141.91 ± 52.57^c^	9236.46 ± 62.00^a^	9093.04 ± 63.42^b^	8099.63 ± 54.84^c^	7905.37 ± 43.79^d^	7606.21 ± 52.65^e^	6481.09 ± 46.53^f^
TPFC*	9011.19 ± 198.98^cd^	9235.9 ± 288.24^ace^	9651.69 ± 314.66^ab^	9633.02 ± 134.52^a^	9172.41 ± 443.96^ad^	9024.89 ± 311.42^bde^	8473.4 ± 414.04^cdf^	8333.35 ± 286.65^f^	8942.1 ± 265.70^cd^
TPLC/ TPFC*	59.53	67.37	84.36	95.88	99.13	89.75	93.30	91.27	59.53

*‘TPLC’ and ‘TPFC’ mean total phenols determined by HPLC analysis (mg/100 g d.w.) and Folin–Ciocalteu assay (mg GAE/100 g d.w.), respectively; ‘TPLC/ TPFC’ means the proportion of total phenols determined by HPLC analysis over total phenols determined by Folin–Ciocalteu assay (%).

On the contrary, the three gallic acid derivatives considerably increased during the first 8 h. There was a 7.14- and 5.09-fold increase for 1,2,3,4,6-pentakis-*O*-galloyl-β-d-glucose and ethyl gallate at 8 h, respectively. After that, they both gradually decreased but remained to be the top among the 10 phenolics (table 2). It should be noted that, among the 10 major phenolics, gallic acid was the only one that kept increasing during the entire thermal treatment, giving a 6.31-fold increase within 24 h. As gallic acid is the common basic unit of 1,2,3,4,6-pentakis-*O*-galloyl-β-d-glucose and ethyl gallate, and both of them stopped increasing after 8 h, we hypothesized that the rise of gallic acid in 8 h might be caused by their depolymerization in the prolonged thermal treatment. It can also be speculated that the increased generation of 1,2,3,4,6-pentakis-*O*-galloyl-β-d-glucose and ethyl gallate within the first 8 h might be caused by the reduction of other unidentified compounds with even higher molecular weight.

As also shown in table 2, total phenols determined by HPLC (TPLC) and Folin–Ciocalteu assay (TPFC) of ATL both increased from 0 to 8 h, and then decreased. Therefore, gallic acid derivatives might be the dominant constituents making up total phenols and influenced by the thermal treatment, which can be validated by the fact that there existed extreme significance (*R*^2^ > 0.842, *p* < 0.01) among 1,2,3,4,6-pentakis-*O*-galloyl-β-d-glucose, ethyl gallate and TPLC. In addition, the proportion of TPLC over TPFC gradually increased from 55.18% in unprocessed ATL to approximately 100% from 4 to 10 h (100.82% at 4 h, 96.83% at 8 h and 95.96% at 10 h), indicating that nearly all phenolics in ATL were converted to the 10 major phenolics during the thermal treatment.

#### 3.2.2. Changes of the 10 major phenolics in *Acer truncatum* flowers

As for ATF, total content of the three gallic acid derivatives (3064.63 mg 100 g d.w.^−1^) were higher than the sum of all six flavonol-3-*O*-glycosides (2222.24) before treatment, indicating that dominant phenolic constituents in unprocessed ATF were different from those in unprocessed ATL. Moreover, flavonol-3-*O*-rhamnosides were no longer the leading compounds of the six flavonol-3-*O*-glycosides as quercetin-3-*O*-galactoside (562.14) and quercetin-3-*O*-arabinopyranoside (326.33) both exhibited considerable levels (table 2). Flavonol-3-*O*-glycosides kept relative steady at the beginning, then significantly decreased to their lowest levels at 24 h, but their declines were much more rapid than those in ATL. As for gallic acid derivatives, 1,2,3,4,6-pentakis-*O*-galloyl-β-d-glucose and ethyl gallate increased from 0 to 2 h in ATF instead of from 0 to 8 h in ATL, giving rise to a 2.18- and 2.87-fold rise, respectively. While 1,2,3,4,6-pentakis-*O*-galloyl-β-d-glucose kept decreasing after 2 h following the pattern in ATL, there was no significant difference among ethyl gallate at 2 h and those with longer thermal treatment. On the other hand, gallic acid still kept increasing during the entire thermal treatment, bringing about a 4.90-fold increase within 24 h. Overall, the dynamic patterns of the 10 major phenolics in ATF were similar to those in ATL, with the reactions in the former being more drastic than those in the latter.

In ATF, TPLC were the highest at 2 h while TPFC were the highest at 1 and 2 h with no significant difference (table 2). The increase of TPLC in ATF was also mainly affected by 1,2,3,4,6-pentakis-*O*-galloyl-β-d-glucose and ethyl gallate as indicated by significant correlations among them (*R*^2^ > 0.686, *p* < 0.05). Meanwhile, the proportion of TPLC over TPFC in ATF also gradually increased from 59.53% to higher than 89% at 2–12 h.

#### 3.2.3. Mechanism underlining the changes of phenolics in *Acer truncatum* leaves and flowers

According to previous studies, thermal treatment could liberate oligomers from their native polymers which are represented as repeating subunits of the oligomers [26]. Moreover, Kim *et al*. [27] reported the enhancement of antioxidant ability of tannic acid after the thermal process and concluded that it was mainly contributed by the increase of pyrogallol and gallic acid during the thermal hydrolysis [28]. Therefore, the large increase of the three gallic acid derivatives in ATL and ATF upon thermal treatment in the present study might indicate drastic depolymerization of gallotannins with higher molecular weight.

**Figure 3.**
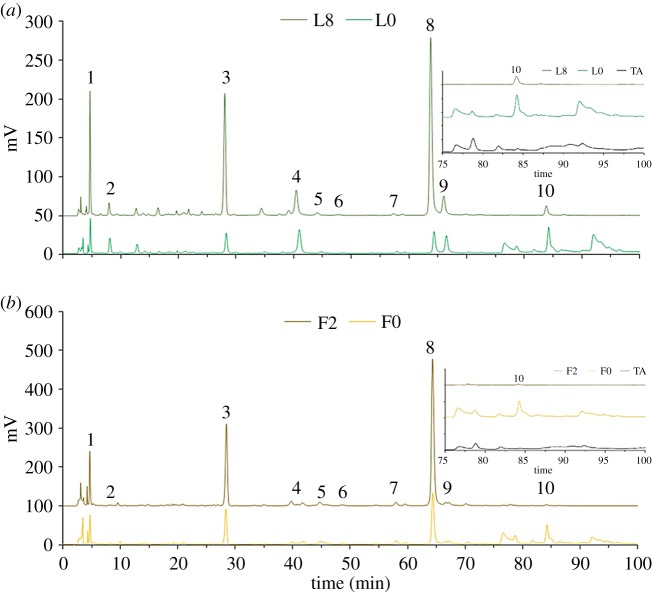
HPLC profiles of ATL and ATF with and without thermal treatment. All chromatographs of ATL (*a*) and ATF (*b*) were obtained under the same HPLC condition described. Insets on the upper right corner of (*a*) and (*b*) are the enlargement of (*a*) and (*b*) between 75 and 100 min, respectively, together with corresponding chromatographs of tannic acid. ‘L0’ and ‘L8’ represents ATL for thermal treatment at 0 and 8 h; ‘F0’ and ‘F2’ represents ATF for thermal treatment at 0 and 2 h.

As shown in figure 3, peaks eluted out between 75 and 100 min in the profiles of unprocessed ATL and ATF totally vanished after the thermal treatment at 8 h for ATL (see dark green curve in figure 3*a*) and at 2 h for ATF (see dark yellow curve in figure 3*b*) except for kaempferol-3-*O*-rhamnoside (compound 10). After comparing the HPLC chromatograph of tannic acid (see black curves in the insets in figure 3*a,b*) with those of unprocessed ATL and ATF, peaks eluted out between 75 and 100 min and their HPLC profiles largely coincided. As reported by Rodríguez *et al*. [29], commercial tannic acid was usually a gallotannins mixture extracted from plants and tended to undergo autodegradation even at room temperature, thus was composed of a set of depolymerized gallotannins and gallates with similar structure and polarity. Therefore, it could be concluded that compounds eluted out between 75 and 100 min in unprocessed ATL and ATF were high polymerization gallotannins which were eventually converted to 1,2,3,4,6-pentakis-*O*-galloyl-β-d-glucose, ethyl gallate and gallic acid. Meanwhile, the main chemical changes during the thermal treatment could be identified as the thermal hydrolysis of gallotannins.

It was reported that the higher the molecule weight and/or polymerization of tannins, the stronger the anti-nutritional effects and the lower the biological activities [30]. Therefore, thermal treatment is a simple and effective way to hydrolyse harmful gallotannins with high molecule weight to improve health benefits of ATL and ATF. Moreover, thermal hydrolysis led to exponential increases of 1,2,3,4,6-pentakis-*O*-galloyl-β-d-glucose, ethyl gallate, and gallic acid, especially in ATL, indicating that it would be an effective preparation method to obtain these three valuable compounds.

### 3.3. Changes of antioxidant activity of the 10 major phenolics

#### 3.3.1. Antioxidant activities of the 10 major phenolics

**Figure 4.**
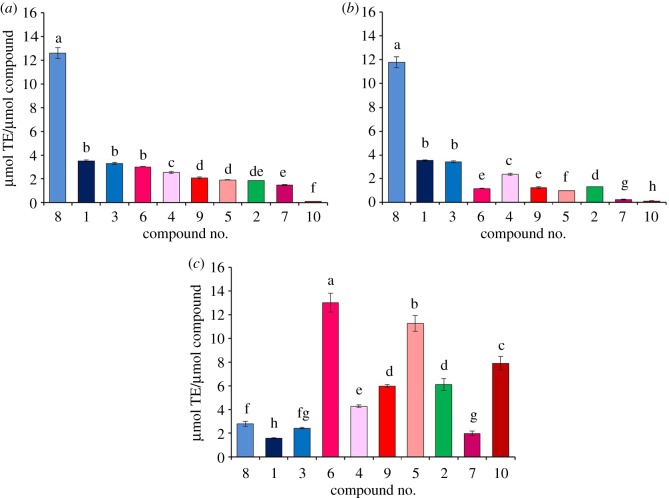
Antioxidant ability of the 10 major phenolics determined with DPPH (*a*), ABTS (*b*) and ORAC (*c*) assays. Compound numbers 1–10 represent the 10 major phenolics occurring in both ATL and ATF, namely gallic acid (1), neochlorogenic acid (2), ethyl gallate (3), myricetin-3-*O*-rhamnoside (4), quercetin-3-*O*-galactoside (5), quercetin-3-*O*-glucoside (6), quercetin-3-*O*-arabinopyranoside (7), 1,2,3,4,6-pentakis-*O*-galloyl-β-d-glucose (8), quercetin 3-*O*-rhamnoside (9) and kaempferol-3-*O*-rhamnoside (10). The order of the 10 major phenolics in the figure was based on the rank of their DPPH• scavenging ability with the highest on the left and the lowest on the right. Different letters means there exists significant difference (*p *< 0.05).

Different phenolics exhibit diverse antioxidant activities depending on their chemical structures, especially numbers of aromatic hydroxyl groups and their positions [31–33]. After characterization of changes of the 10 major phenolics during the thermal treatment, we further determined their antioxidant activities with DPPH, ABTS and ORAC assays. As shown in figure 4*a*, the rank order of DPPH• scavenging abilities of the 10 major phenolics was as follows: gallotannin (8)≫gallates ( 1, 3)  > flavonol-3-*O*-glycosides (6, 4, 9, 5, 7 and 10) and caffeoylquinic acid (2). Meanwhile, their ABTS^+^• scavenging abilities showed a similar rank order except myricetin-3-*O*-rhamnoside (4) and neochlorogenic acid (2) which exhibited a little higher ability (figure 4*b*).

For gallic acid derivatives, polymerization was an antioxidant increasing step based on molar concentration as 1,2,3,4,6-pentakis-*O*-galloyl-β-d-glucose (8) showed a significant higher antioxidant ability (3.59-fold for DPPH, and 3.38-fold for ABTS) than that of gallic acid (1). However, a contrary result was obtained based on mass unit as one 1,2,3,4,6-pentakis-*O*-galloyl-β-d-glucose consisted of five gallic acid units while it exhibited less than fivefold higher DPPH or ABTS value comparing with gallic acid. As for flavonol-3-*O*-rhamnosides, their DPPH and ABTS values were ranked as myricetin-3-*O*-rhamnoside (4) > quercetin-3-*O*-rhamnoside (9) > kaempferol-3-*O*-rhamnoside (10), exhibiting that the more hydroxyl groups on their ring B, the higher DPPH• and ABTS^+^• scavenging activity they possessed. The same pattern was observed in the study reported by Tabart *et al*. [34], as they determined the DPPH value of myricetin-3-*O*-rhamnoside was higher than that of quercetin-3-*O*-rhamnoside, and both of them were higher than that of kaempferol-3-*O*-glycosie. Among quercetin-3-*O*-glycosides, quercetin-3-*O*-glucoside (6) exhibited the highest DPPH value while quercetin-3-*O*-rhamnoside (9) exhibited the highest ABTS value. They both showed a higher scavenging ability than quercetin-3-*O*-galactoside (5), while the quercetin-3-*O*-arabinopyranoside (7) exhibited the lowest in both assays.

Antioxidant ability of the 10 major phenolics determined with ORAC assay (figure 4*c*) was quite different from those with DPPH or ABTS (figure 4*a,b*). The rank order could be summarized as flavonol-3-*O*-glycosides (9, 6, 5, 10, 4 and 7) and caffeoylquinic acid (2) > gallotannins (8) > gallates (1, 3), except that quercetin-3-*O*-arabinopyranoside (7), one of the flavonols-3-*O*-glycosides, was lower than each of the gallotannins and gallates, i.e. 1,2,3,4,6-pentakis-*O*-galloyl-β-d-glucose (8) and ethyl gallate (3).

Gallic acid derivatives, which were significantly higher in DPPH and ABTS activities, exhibited lower ORAC values than flavonol-3-*O*-glycosides and caffeoylquinic acids. Tabart *et al*. [34] reported that gallic acid possesses a relative similar activity in DPPH and ORAC assays while all flavonols, flavan-3-ols and flavanons exhibited a much higher ORAC value than DPPH value, which supported the results of this study. In addition, myricetin-3-*O*-rhamnoside (4), with three hydroxyl groups on its ring B, exhibited the lowest ORAC value while kaempferol-3-*O*-rhamnoside (10), with only one hydroxyl group on its ring B, exhibited the highest value, which was very different from the patterns in DPPH and ABTS assays. A few studies investigated the ORAC values of kaempferol, quercetin and myricetin, giving a concordant rank order as kaempferol > quercetin > myricetin [34–36]. Thus, the conclusion could be drawn that the number of hydroxyl groups on the ring B exhibited a negative impact on ORAC values for myricetin, quercetin and kaempferol, as well as their 3-*O*-glycosides. On the other hand, the ORAC order of quercetin-3-*O*-glycosides was quercetin-3-*O*-rhamnoside (9) > quercetin-3-*O*-glucoside (6) > quercetin-3-*O*-galactoside (5) > quercetin-3-*O*-arabinopyranoside (7), which was the same with that in ABTS and similar with that in DPPH, indicates that these glycosides exerted diverse impacts on their quercetin aglycone in the same order in all three antioxidant assays.

It is known that radicals are mainly quenched by two chemical principles, transfer of either a hydrogen atom or a single electron to convert the radicals to stable forms [37–39]. DPPH and ABTS assays are based on the transfer of a single electron, and measure the electron-donating capacity of antioxidants to reduce the radicals (DPPH• or ABTS^+^•). As for the ORAC method, antioxidants slow the loss of fluorescence by quenching the peroxyl radicals produced by AAPH via transfer of hydrogen atom or radical addition. The difference between these two mechanisms might elucidate the huge variation between the rank order of the 10 major phenolics in DPPH and ABTS and ORAC assays. Furthermore, it revealed that the 10 major phenolics, as main phenolic composition in both ATL and ATF, exhibited not only a high electron-donating but also a high chain-breaking capacity.

#### 3.3.2. Antioxidant contributions of the 10 major phenolics to total antioxidant activity in *Acer truncatum* leaves and flowers

Apart from contents of the 10 major phenolics and their individual antioxidant activities, contributions of them to the total antioxidant activities were also evaluated. Within a single sample, the calculated contribution of each phenolic compound was obtained by multiplying its content measured by HPLC and the corresponding DPPH, ABTS or ORAC value (Trolox equivalent). The sum of the contributions of all 10 phenolics in the single sample was defined as the total calculated antioxidant activity.

**Figure 5.**
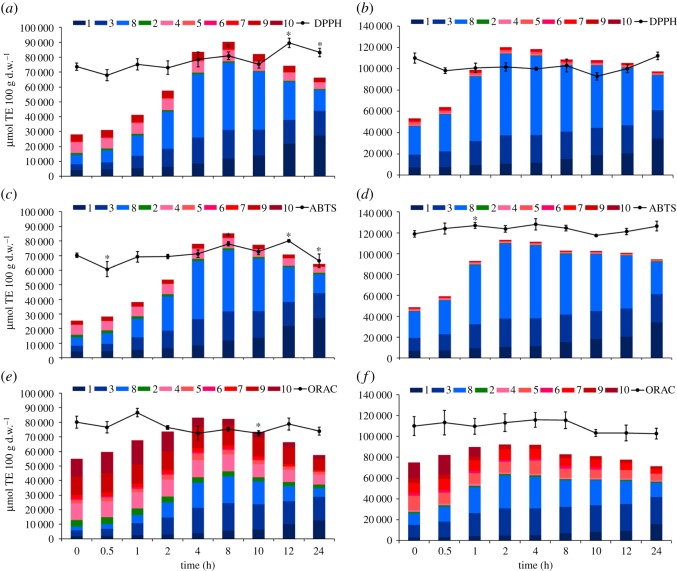
Antioxidant contributions of the 10 major phenolics (colour columns) to the total antioxidant activity (dark lines) in ATL (*a,c,e*) and ATF (*b,d,f*). Columns of gallic acid derivatives were coloured with cold (blue), columns of flavonols-3-*O*-glycosides were coloured with warm (red) and the column of caffeoylquinic acid was coloured in green. Asterisk (*) means there exists significant difference between total antioxidant activity (DPPH, ABTS or ORAC) of unprocessed ATL or ATF and those after thermal treatment.

As shown in figure 5, contributions of the 10 major phenolics (colour columns) to the total measured DPPH (figure 5*a,b*), ABTS (figure 5*c,e*) and ORAC (figure 5*d,f*) values (dark lines) ranged from 35.15% (DPPH for ATL at 0 h) to 123.93% (ORAC for ATL at 4 h). Within most of the treatments, the proportion was higher than 50%, further confirming the dominant contribution of the 10 major phenolics. In addition, all contribution proportions (total calculated antioxidant activity/total measured antioxidant activity) for DPPH, ABTS and ORAC values were lower at the beginning and end period within the 24 h treatment for both ATL and ATF. The HPLC-undetectable gallotannins with high polymerization was responsible for the low contribution proportion at the beginning of the treatment while the HPLC-undetectable hydrolysis products other than the 10 major phenolics was responsible for that at the end period of the treatment.

During the entire treatment, gallic acid derivatives always made an important contribution to the total activities of both measured and calculated, accounting for more than 50% of the total calculated activity in ATL, and more than 90% of that in ATF with both DPPH and ABTS assay (see blue colour columns in figure 5*a–d*). On the contrary, they made a relatively lower contribution to ORAC values as they accounted for 15.08%–59.99% in ATL and 34.79%–73.39% in ATF from 0 to 24 h (see cold colour columns in figure 5*e,f*). On the other hand, flavonol-3-*O*-glycosides and caffeoylquinic acid made little contribution to the total DPPH and ABTS values (see warm colour and green columns in figure 5*a–d*). However, they made a 76.43% and 63.11% contribution to ORAC values in unprocessed ATL and ATF and gradually decreased to 35.20% and 20.86% at 24 h, respectively (see warm colour and green columns in figure 5*e,f*).

As shown in figure 5*a–c,e*, there is a certain period for DPPH in ATF and for all three assays in ATL when the contribution proportions were found to be even larger than 100%. It might reveal that there is specific compound(s) inhibiting the antioxidant capacity of phenolics in ATL and ATF extracts and thus limiting the total antioxidant activity, or certain phenolics in ATL and ATF showed an antagonistic effect in total antioxidant activity. Palafox-Carlos *et al*. [40] reported that isolated gallic, protocatechuic, chlorogenic and vanillic acids from mango cultivar ‘Ataulfo’ showed high antioxidant activities against DPPH•. However, the antagonistic effect was observed if gallic acid was combined with vanillic acid, or in the case that protocatechuic was combined with chlorogenic and vanillic acids. Moreover, Heo *et al*. [41] found that the addition of chlorogenic acid to cyanidin, peonidin-3-*O*-glucoside, quercetin and quercetin-3-*O*-galactoside caused a decrease of DPPH values compared with their total calculated antioxidant capacity. A number of phenolics mentioned above, including gallic and neochlorogenic acid, and quercetin-3-*O*-galactoside were important phenolic constituents of ATL and ATF. Therefore, we assume that certain phenolics in ATL and ATF exhibited an antagonistic effect to the total antioxidant activity, resulting in that there are several ATL and ATF samples whose total calculated activity were higher than their total measured activity as shown in figure 5*a–c,e*.

Overall, the thermal treatment possessed a positive effect on gallic acid derivatives with aspect to both their contents and antioxidant contributions (see cold colour columns in figure 5). However, total measured antioxidant activity did not remarkably increase (see dark lines in figure 5) partially owing to the fact that flavonol-3-*O*-glycosieds, caffeoylquinic acids and their antioxidant contributions gradually decreased under the treatment (see warm colour and green columns in figure 5). Based on this conjecture, further work should be focused on the isolation process so that total antioxidant activity could be improved more through an accurate hydrolysation of polymerized gallotannins alone.

## 4. Conclusion

The established thermal treatment method was effective to achieve massive gallic acid derivatives (i.e. 1,2,3,4,6-pentakis-*O*-galloyl-β-d-glucose, ethyl gallate and/or gallic acid), which exhibited higher antioxidant ability and nutritional effect compared with high polymerization gallotannins in unprocessed ATL and ATF. Although thermal treatment significantly decreased flavonol-3-*O*-glycosides, total phenols and antioxidant ability in ATL and ATF still significantly increased. Thermal hydrolysis were responsible for the variation of phenolic composition and antioxidant contribution.

## Supplementary Material

Supplementary Figures and Table

## Data accessibility

All datasets supporting this article have been uploaded as the electronic supplementary material and this article does not contain any other additional data.
