# Supplementary material for: Effects of thermal treatments on 10 major phenolics and their antioxidant contributions in Acer truncatum leaves and flowers
Source: R Soc Open Sci. 2018 Jun 27;5(6):180364. doi: 10.1098/rsos.180364 (PMC6030319; doi:10.1098/rsos.180364)
Supplement: Supplementary Figures and Table [file rsos180364supp1.docx]

Effects of thermal treatments on ten major phenolics and their antioxidant contributions in *Acer truncatum* leaves and flowers

Lingguang Yang^a^, Peipei Yin^a^, Chi-Tang Ho^b^, Miao Yu^a^, Liwei Sun^a^*, and Yujun Liu^a^*

a. National Engineering Laboratory for Tree Breeding, College of Biological Sciences and Biotechnology, Beijing Forestry University, Haidian District, Beijing, China.

b. Department of Food Science, Rutgers University, New Brunswick, NJ, USA.

**Keywords:** *Acer truncatum* leaves and flowers; thermal treatment; phenolics; antioxidant contribution; UPLC-DAD-QTOF-MS/MS analysis.

*Author for correspondence Liwei Sun (lsun2013@bjfu.edu.cn); Yujun Liu (yjliubio@bjfu.edu.cn).

†Present address: National Engineering Laboratory for Tree Breeding, College of Biological Sciences and Biotechnology, Beijing Forestry University, Haidian District, Beijing, China.

Table S1. Regression equations, determination coefficients and linear ranges of the ten major phenolics from ATL and ATF.

| Phenolics (peak no.) | Regression equation^*^ | *R*^2^ | Linear range  (mg/mL) |
| --- | --- | --- | --- |
| gallic acid (1) | y = 3 × 10^7^x - 133926 | 0.9995 | 0.043-0.172 |
| neochlorogenic acid (2) | y = 2 × 10^7^x - 39036 | 0.9983 | 0.008-0.033 |
| ethyl gallate (3) | y = 3 × 10^7^x - 33353 | 0.9996 | 0.010-0.040 |
| myricetin-3-*O*-rhamnoside (4) | y = 1 × 10^7^x - 67256 | 0.9987 | 0.014-0.057 |
| quercetin-3-*O*-galactoside (5) | y = 1 × 10^7^x - 11301 | 0.9997 | 0.005-0.020 |
| quercetin-3-*O*-glucoside (6) | y = 2 × 10^7^x - 50852 | 0.9986 | 0.006-0.023 |
| quercetin-3-*O*-arabinopyranoside (7) | y = 8 × 10^6^x - 2171 | 0.9997 | 0.003-0.013 |
| 1,2,3,4,6-pentakis-*O*-galloyl-β-d-glucose (8) | y = 2 × 10^7^x - 50259 | 0.9988 | 0.004-0.017 |
| quercetin 3-*O*-rhamnoside (9) | y = 1 × 10^7^x - 96683 | 0.9999 | 0.024-0.010 |
| kaempferol-3-*O*-rhamnoside (10) | y = 2 × 10^7^x - 35659 | 0.9995 | 0.006-0.027 |

*y is the peak area of chromatography, and x is the concentration of individual standards (mg/mL);

*R*^2^ is the determination coefficient of the corresponding equations.


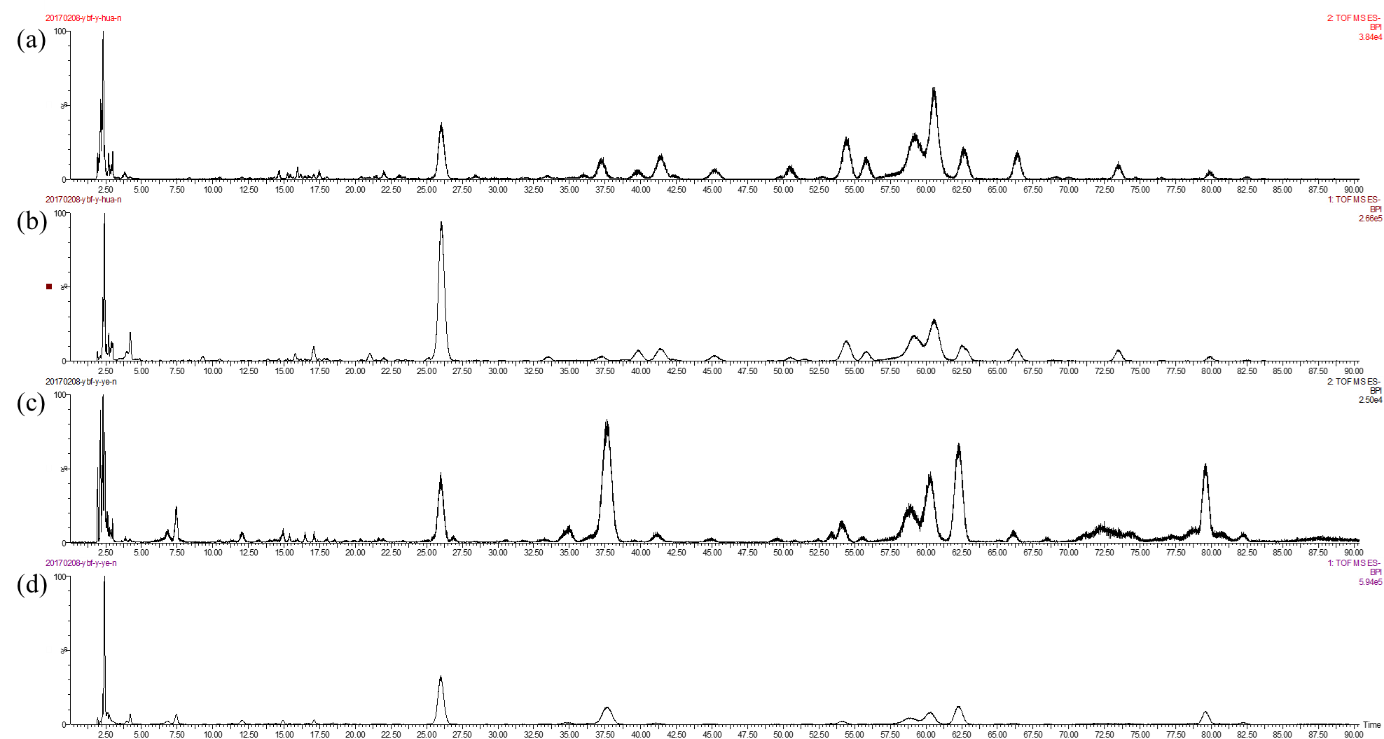


Fig. S1. Base Peak Chromatogram of MS and MS/MS spectra of ATF (a, b, respectively) and ATL (c, d, respectively) extracts obtained under the same UPLC conditions.


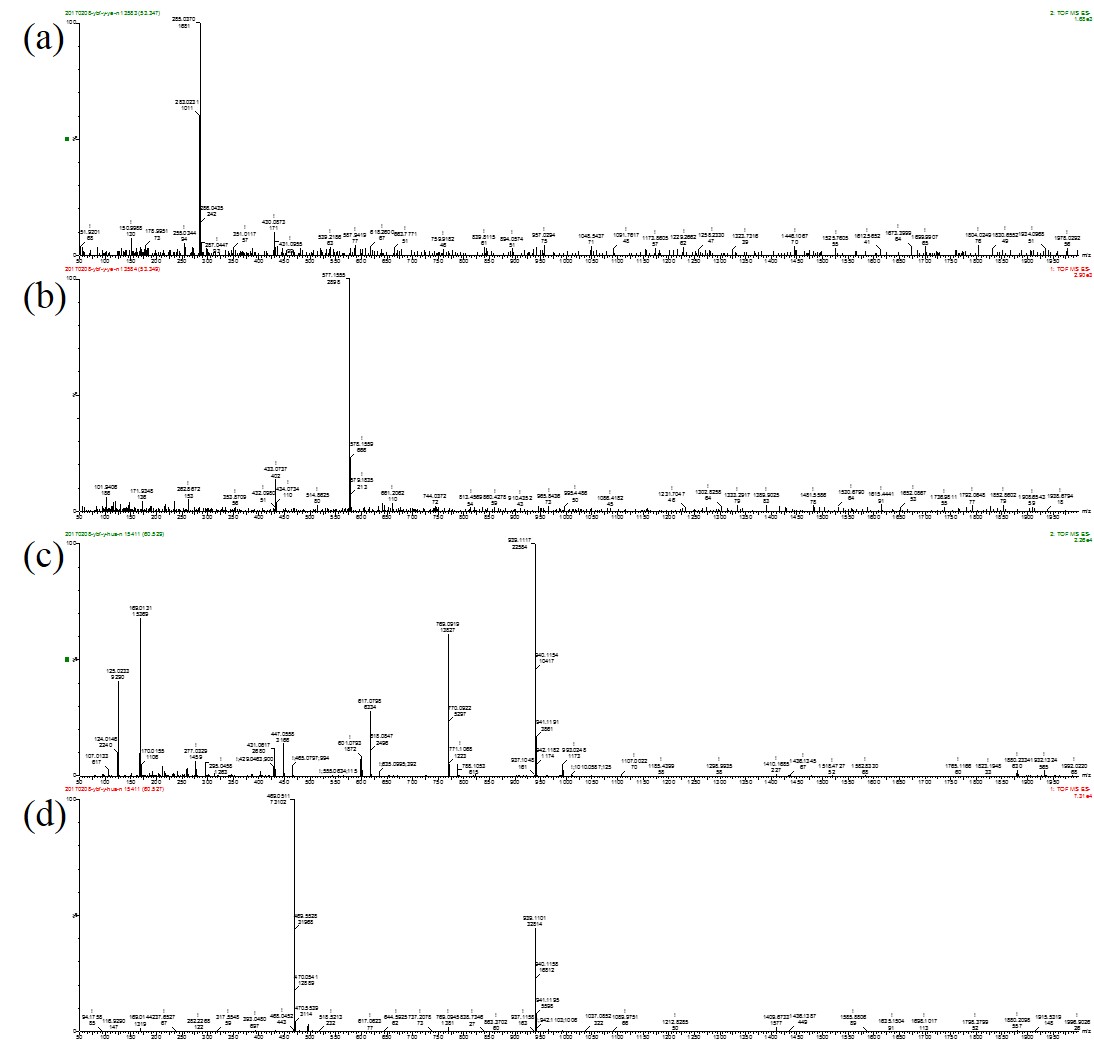


Fig. S2. MS and MS/MS for the identification of kaempferol-3,7-*O*-dirhamnoside (a, b, respectively), 1,2,3,4,6-pentakis-*O*-galloyl-β-d-glucose (c, d, respectively).
